# Implementation, Outputs, and Cost of a National Operational Research Training in Rwanda

**DOI:** 10.5334/aogh.2933

**Published:** 2020-08-05

**Authors:** Jackline Odhiambo, Ann C. Miller, Naome Nyirahabimana, Loise Ng’ang’a, Fredrick Kateera, Bethany L. Hedt-Gauthier

**Affiliations:** 1 Nyanam International, Kisumu, KE; 2 Department of Global Health and Social Medicine, Harvard Medical School, Boston, US; 3 Partners In Health-Inshuti Mu Buzima, Kigali, RW

## Abstract

**Background:**

Training and mentorship in research skills are essential to developing a critical mass of researchers in low- and middle-income countries (LMICs). However, reporting on the details of such training programs, especially regarding the cost of the training, is limited.

**Objectives:**

This paper describes a year-long operational research training and mentorship course in Rwanda, implemented between 2013 and 2017.

**Approach:**

We describe motivations for the design of the Intermediate Operational Research Training Course (IORT) across four iterations. We also report outputs, evaluate trainee experiences, and estimate training and mentorship costs.

**Findings:**

Of the 132 applicants to the course, 55 (41.7%) were selected, and 53 (96.4%) completed the training. The ratio of female-to-male trainees in the course increased from 1:8 in 2013 to 1:3 in 2017. Trainees developed and co-first-authored 28 research manuscripts, 96.4% (n = 27) of which are published in peer-reviewed journals. For the 15 trainees who completed the post-course evaluation, 93.3% and 86.7% reported improvement in their research and analytical skills, respectively. The median cost per trainee to complete the course was US$908 (Range: US$739–US$1,253) and per research project was US$2,708 (US$1,748–US$6,741). The median annual training delivery and mentorship cost was US$47,170 (US$30,563–US$63,849) for a course with a Rwanda-based senior mentor, junior mentor, and training coordinator. The total essential cost for a year-long IORT course with 16 trainees co-leading eight research projects and mentored by two senior and four junior mentors was US$101,254 (US$73,486–US$157,569).

**Conclusion:**

We attribute the high course completion rates, publication rates, and skills acquisition to the learning-by-doing approach and intensive hands-on mentorship provided in the course. IORT was costly and funded through institutional resources and international partnerships. We encourage funders to prioritize comprehensive research capacity-building initiatives that provide intensive mentorship as these are likely to improve the pool of skilled researchers in LMICs.

## Introduction

Training and mentorship in core biomedical research knowledge and skills are essential for building individual and institutional research capacity in low- and middle-income countries (LMICs) [1,2,3]. Capacitated LMIC researchers can produce research that is relevant to national priorities, link research with policy and practice, and negotiate equitable international research collaborations [1,4,5]. Efforts to improve national health research capacity in LMICs, led by academic institutions and non-governmental organizations, have steadily increased [6,7,8]. Currently, LMIC authors lead approximately half of health-related publications about LMICs [9,10].

Within sub-Saharan Africa, research output and the reported progress in authorship dynamics reflect improvements in only a small subset of countries, masking the continental inequity in research capacity [6,11]. To bridge this gap, Rwanda is one of the sub-Saharan African countries prioritizing investments in local research capacity and equitable research collaborations [12,13,14]. Rwanda’s fourth health sector strategic plan recognizes the need for evidence for decision making and to strengthen the national health research system [12]. This strategic plan promotes strengthening local and international research collaborations and establishing a fund for operational research in health facilities [12]. The Rwanda Ministry of Health (RMoH) research guidelines require evidence for individual or infrastructural research capacity building with international research collaborators [14]. These research guidelines also protect research time for Rwandan health workers and aim to increase research participation and leadership by Rwandans.

Partners In Health Rwanda (PIH/Rwanda) is an international non-profit organization that has supported the RMoH in health care delivery since 2005. PIH/Rwanda began a comprehensive research capacity building initiative in rural Rwanda in 2012 [15]. As part of this initiative, PIH/Rwanda implements three tiers of incrementally intensive research capacity building courses. The foundational course is a 10-lecture Introduction to Research Methods course [16]. This course introduces core epidemiological and biostatistical concepts to cultivate a research culture and increase consumption of scientific literature. The mid-level course, Intermediate Operational Research Training (IORT) course [17], was modeled after the World Health Organization’s (WHO) Structured Operational Research Training Initiative (SORT IT) course [18]. IORT builds core research skills through research project leadership. The third-level training strengthens research leadership and independence by providing scholarships for masters or PhD degrees in Rwandan universities through various partnerships [15]. This paper shares our experiences with IORT courses offered between 2013 and 2017, specifically with regard to cost and implementation process. We hope this description catalyzes efforts towards nationalization of operational research capacity building in LMICs.

## Training design

Research training courses in LMICs vary in terms of their structure, duration, and the research competencies they cover [2]. The availability, type, and intensity of mentorship trainees receive also differs [2]. For IORT, we aimed to train and mentor a cadre of Rwandan researchers who can lead research projects from the initial idea to the publication of research findings. We describe each of the course elements, providing details on our motivations for each decision.

### Training approach, structure, duration, and competencies

IORT is a learning-by-doing course that uses a deliverable-driven model to teach research skills. Learning-by-doing is a hands-on training approach in which the learner interacts with their environment or project to enable learning [19]. This approach exemplifies ‘doing’ beyond ‘seeing’ and ‘hearing’ during learning. Thus, in IORT, trainees learn research skills by implementing research projects that are of consequence to service delivery and that are publishable. The deliverable-driven model, which breaks down the research process into smaller, manageable components (deliverables), enhances this practical approach. Deliverables help deconstruct the research process to new learners, focusing on smaller milestones that culminate to a research paper. Each milestone has clearly defined outputs and timelines to track learning and progress.

**Figure 1:**
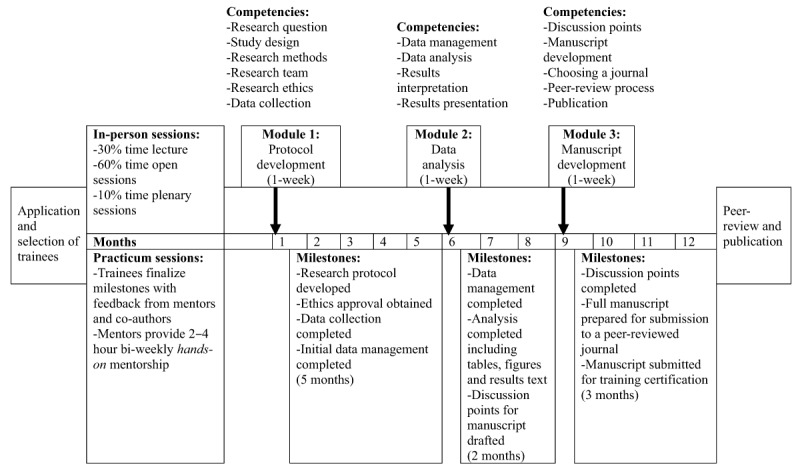
Design of Partners In Health/Rwanda’s Intermediate Operational Research Training in 2016 and 2017.

IORT consists of alternating in-person sessions and practicum sessions (Figure 1). During in-person sessions, we implement short lectures on research concepts and skills. We then give trainees time to apply the concepts to their projects. This process reinforces skills acquisition and confidence, and ensures that trainees receive adequate support to effectively implement their research projects [20,21]. Trainees present their work to their peers for feedback in plenary sessions, strengthening their communication, peer learning, and public engagement skills. Plenary sessions also encourage a culture of critical appraisal of research. The practicum periods follow each in-person session and enable trainees to complete milestones with ongoing mentorship and active engagement from their research authorship team. Trainees who complete each milestone in time for the next in-person session continue participating in the course.

Each course is 8–12 months long. We shifted the frequency and duration of in-person sessions from seven two-day sessions in the first two cohorts to three one-week sessions in the latter courses (Table 1). We found that the short-length high-frequency meetings facilitated stepwise research learning in a context of limited research exposure [17]. However, the frequent meetings disrupted the trainees’ full-time jobs, required more planning, and became cost and time prohibitive when mentors had to travel internationally to facilitate the course [17]. The three one-week sessions structure enabled focused research time with more in-person mentorship and reduced workload in the practicum periods (Figure 1).

**Table 1:** Iterations of the PIH/Rwanda Intermediate Operational Research Course.

Area	The first course, 2013	The second course, 2015	The third and fourth course, 2016/2017

**Training structure, duration, and competencies**	A deliverable-driven training model implemented over 8–12 months, using learning by doing teaching approach and intensive hands-on mentorship. The course has in-person sessions (lectures, open sessions, and plenary sessions) and practicum periods. Competencies covered include developing research protocol, research ethics, study design, research team, data collection and management, data analysis and interpretation, manuscript writing, dissemination. Trainees complete all milestones to stay in the program and to receive certificate.		
	7 2-day modules, every 4–6 weeks, with 7 milestones. The first 6 milestones completed prior to the next training session. The final manuscript submitted to a peer-reviewed journal but had no specific deadline. All course mentors were Rwanda-based.		3 6-day modules, every 2–5 months, with 3 milestones. A longer gap between Modules 1 and 2 allowed for ethical review and data collection. Final manuscript submitted to a peer-reviewed journal by a set deadline. All course mentors were non-Rwanda-based. Introduced peer-review lecture and study site dissemination before paper publication.
**Mentorship and follow-up**	All trainees receive hands-on in-person mentorship during course delivery and practicum, supplemented by ongoing virtual mentorship through emails and skype meetings.		
	2 course mentors and 1 junior mentor. The course mentors supported all 5 research teams for Modules 1–6 and paired with 2–3 teams for Module 7. The junior mentor supported all projects.	2 course mentors, 6 project mentors^‡^ and 3 junior mentors. Course mentors supported all 7 projects together throughout all modules. Each junior mentor supported 2–3 projects. Project mentors mentored 1–2 projects each but did not attend training sessions.	2 course mentors, 4 project mentors and 5 junior mentors. Course mentors supported 4 projects each. Project mentors and junior mentors supported 1–2 projects each. Project mentors did not attend training sessions.
**Selection of trainees and research projects**	A selection committee including institutional leaders and training mentors who selected district-based program and clinical staff. Trainees implemented quantitative retrospective projects that could be completed within 12 months of course implementation. We hired and trained data collectors for trainee projects. Trainees completed data analysis using Stata. PIH/Rwanda provided research administrative support.		
	Trainees applied to the program with a research proposal.	Trainees selected a research topic from a list of research topics submitted by project mentors	
	Trainees supervised data collection with support from junior mentors. Trainees led data cleaning and analysis.		Junior mentors supervised data collection and initial data management. Trainees completed data management and led data analysis. Trainees included post-graduate students and university lecturers.
**Training and research project costs**	All trainees received full scholarship excluding per-diems discouraged by organizational policies. Research costs included publication, conferences, data collection, software, communication, and travels.		
	Each project received US$4000 and trainees managed the budget to cover research costs and trainee supplies and communication.	Each project received US$3000 and trainees managed the budget to cover research costs and trainee supplies and communication.	No project was assigned a specific budget. Research costs were centrally managed for all projects.
**Training evaluation**	Participants completed milestones and submitted their papers to peer-reviewed journals.		
			Participants completed course evaluation.

^‡^ Project mentors were principal investigators with active protocols featured in the second and third courses only. They donated research topics/data to the training; PIH/Rwanda: Partners In Health/Rwanda.

IORT covers research competencies in protocol development, data analysis, and manuscript development. Under protocol development, trainees learn how to craft a research question, select appropriate study design and research methods, build a research team, and collect data while observing research ethics in the process. During data analysis, trainees build their skills in data management, data analysis, results interpretation, and presentation. In manuscript development and publication, trainees identify discussion points from their results, develop their manuscript, learn how to choose a journal for publication, and learn how to manage the peer review to publication process (Figure 1).

### Intensive hands-on mentorship and follow-up

Mentorship is key for a new researcher’s success. Adequate mentorship is needed for research trainees to produce quality research outputs [22,23,24]. Mentorship takes different forms, either *hands-off* or *hands-on* [22]. In *hands-off* mentorship, mentees only receive technical advice from mentors. In *hands-on* mentorship, mentees receive direct technical assistance in writing their research proposal, analyzing their data, and writing up their results for publication [22]. For the IORT, we provided intensive hands-on mentorship that prioritized in-person one-to-one mentorship during training and practicum periods. We supplemented the in-person mentorship with virtual mentorship through Skype calls and e-mail communication, including feedback on trainees’ research materials.

To avail robust mentorship, we engaged three types of mentors: senior course mentors, junior mentors/research assistants, and project mentors. The senior course mentors had extensive research experience with backgrounds in biostatistics, epidemiology, and public health. They led both course delivery and course mentorship. Each supported a maximum of four research projects. To date, all senior course mentors in IORT have been non-Rwandans who were either Rwanda-based or had lived in Rwanda for at least two years. Notably, local mentors are best suited to ensure training program ownership and increase the sustainability of the investments for national research capacity [23,24]. This is because local mentors demonstrate the reality of a research career locally and improve the relevance of research projects to local priorities [23,24]. They also reduce the course delivery costs (for example in regards to international travel costs) that can be prohibitive in nationalizing these training programs [23,24]. However, deploying Rwandan researchers as senior course mentors remains a challenge, partly because those with the experience to fill this role have lacked the protected time required for the intensive hands-on mentorship.

The junior mentors included a pool of Rwandan and non-Rwandans with research backgrounds ranging from recent undergraduates with some research and statistics experience to global health practitioners with master’s degrees in public health. Each junior mentor supported one to three projects, depending on their availability and experience. Similar to senior course mentors, the junior mentors provide real-time hands-on mentorship during in-person training sessions and the practicum period. Junior mentors also received coaching from the senior mentors to grow their research and mentorship skills. We hope to increase the pool of Rwanda-based mentorship team through nurturing the junior mentors to become senior mentors in future iterations of this course.

We introduced project mentors in the 2015 course due to ethical review delays (delays lasted eight months in our 2013 course) and high review costs (US$1,400 per research proposal). These challenges have also been reported elsewhere in LMICs [25,26]. Project mentors were local and international principal investigators with active health research projects in Rwanda. They were also affiliated with different programs implemented or supported by PIH/Rwanda. These mentors contributed research topics to the course from their active protocols, in part to fulfill their RMoH obligations in research capacity building [14]. In addition, they covered research project costs such as for ethical reviews and data collection. They also provided mentorship specifically in the practicum periods as their attendance of in-person training sessions was voluntary. Often, the research staff in the project mentors’ research team served as junior mentors for the associated training project. As seen elsewhere, we found a pre-training orientation and post-training reflection for project mentors useful for sharing training objectives, mentorship expectations, tips, and experiences [23].

Our mentorship process respected trainee leadership of the research project and allowed for growth of both trainees and mentors. With each milestone, the trainees led the drafting of the initial research protocol, data analysis, and manuscript writing. Trainees then received and addressed feedback from all mentors, beginning with junior mentors. We used an iterative process for review, within set timelines, until trainees and their mentors agreed that a drafted research document was ready for feedback from the authorship team. Because of the intensive contribution of mentors in the research process, all mentors formed part of the trainee’s authorship team, enhancing both their research careers and leadership skills [27].

### Selection of trainees

During course application, trainees stated their research background and interests and provided a letter of support from their employer or supervisor. Because all trainees were full time health or academic professionals, we selected trainees jointly with their institutional leaders. We selected trainees based on three criteria. First, the senior course mentors ranked trainees’ readiness to complete the IORT course based on previous research experiences and training. Second, the project mentors ranked trainees’ existing or potential links to the program or research proposed. Third, the employer or work supervisor ranked applicants from their organization based on their organizational priorities for research training. The contribution of all stakeholders in the trainee selection enhanced stakeholder commitment to the course and secured institutional support for trainees to both attend the training and to lead research within their organizations post-training.

Because of limited funding and mentorship resources, we paired trainees on each research project to train more individuals while maintaining a reasonable number of research projects. Pairing facilitated peer-to-peer learning and engendered research partnerships across institutions. However, training teams are best limited to two individuals per project to enable tangible leadership contributions to the research project. Our trainees published as joint first authors.

### Selection of research projects

The selection of research projects in the course shifted from trainees’ self-identified topics in 2015 to trainees selecting topics from a list submitted by the project mentors in later years (Table 1). Self-identified topics enhanced project ownership and ensured that research topics linked closely to trainees’ work. For these reasons, we prefer that trainees identify their own research topics. However, due to the time and cost implication for ethical reviews, we continue to use suggested research topics that are nested within existing approved studies. With this approach, we also shifted from trainees applying to the course with research partners to pairing trainees strategically across institutions and backgrounds. We recommend clustering of research topics under broader themes such as pediatrics, surgery, or non-communicable diseases. This helps trainees to focus on a thematic area of interest. In addition, having at least two projects per theme enhances peer collaboration between trainee teams and promotes focused mentorship as senior course mentors support trainees in their thematic area of interest.

### Course evaluation

Trainees in the 2016 cohort completed a course evaluation. We conducted pre-course surveys with the 36 applicants and mid-course surveys with the 15 trainees. All applicants completed a semi-structured questionnaire about their research background and motivation for the course. The trainees completed the mid-course survey using Kirkpatrick’s first level of training evaluation model to assess their reactions to course materials, delivery, and outcome [28].

We analyzed qualitative responses in both evaluations thematically. We used a Likert scale with scores of strongly agree (score = 4), agree (score = 3), disagree (score = 2) and strongly disagree (score = 1) to measure trainee reactions. To measure training pace in the mid-course survey, we applied the scale as too fast (score = 3), just right (score = 2), and too slow (score = 1). We calculated the median Likert score and frequency of respondents with each median score.

### Training and mentorship costs

We collected information on annual programmatic training and research project expenditures from research, finance, human resources, and procurement teams. We collected both cash and in-kind expenditures per course, as well as the corresponding number of training participants and research projects. We grouped expenditures into three categories: trainee costs, research project costs, and training delivery and mentorship costs (Table 2). We converted per course costs from Rwandan Francs to United States Dollars using an annual average conversion rate. Cost estimates did not account for inflation rate, which averaged 0.36% over the time period and would have minimal effect on the cost estimates [29]. We then calculated the average cost per cost item (e.g. trainee travels) per course to estimate unit costs. We report the median and range unit costs. We also estimated the median and range costs for a year-long, three one-week sessions course, with 16 trainees paired to lead eight research projects and supported by two senior course mentors and four junior mentors.

## Course outputs and trainee experiences

### Course outputs

Between 2013 and 2017, 132 candidates applied for the IORT courses, 55 (41.7%) of whom were selected based on their research background, experience with chosen research project, and institutional training priorities (Table 3). Of those 55 admitted trainees, 53 (96.4%) completed the training by participating in the training sessions and completing and submitting their research project to a journal for publication. The ratio of female-to-male trainees in the course increased from 1:8 in 2013 to 1:3 in 2017. In the first two courses, our trainees were primarily clinical and program staff from PIH/Rwanda and district level RMoH. In the latter two courses, we admitted trainees from the University of Rwanda – College of Medicine and Health Sciences (33.3%, n = 5 in 2016) and the Rwanda Biomedical Center (20.0%, n = 3 in 2017). The research topics in the courses covered diverse health issues in Rwanda, such as infectious diseases (3 papers), maternal and child health (7 papers), non-communicable diseases (6 papers), surgery (9 papers), and pharmacy (3 papers) (Table 4).

**Table 2:** Cost items for IORT cost estimation.

Trainee costs	Research project costs	Training delivery and mentorship costs

•Travels to training venue	•Data collection	•Training venue
•Meals during training	•Training of data collectors	•Administrative support
•Room and board	•Ethics review costs	•Staff time - Training design and logistics - Developing training materials - Course facilitation - Mentorship
• Research communications	• Conference attendance^†^	
• Project supplies: - Data analysis software - Flash drives - Laptops^†^	•Research meetings	
	•Publication fees^†^	

^†^ We relied on existing resources to meet these costs e.g. using trainees’ own laptop or borrowed laptop from PIH/Rwanda and requesting for scholarships for international conference attendance outside Rwanda and fee waivers for publication.

**Table 3:** Description of trainees and training outputs in the IORT course, n (%).

	2013	2015	2016	2017	Total

**Total applications**	**24**	**28**	**36**	**44**	**132**
Male	23 (95.8)	20 (71.4)	30 (83.3)	28 (63.6)	101 (76.5)
Female	1 (4.2)	8 (28.6)	6 (16.7)	16 (36.4)	31 (23.5)
**Selected applicants**	10 (41.7)	13 (46.4)	16 (44.4)	16 (36.4)	55 (41.7)
**Applicants completing the training**	9 (90.0)	13 (100.0)	15 (93.8)	16 (100.0)	53 (96.4)
Male	8 (88.9)	11 (84.6)	11 (73.3)	12 (75.0)	42 (79.2)
Female	1 (11.1)	2 (15.4)	4 (26.7)	4 (25.0)	11 (20.8)
**Occupation of trainees**					
Clinical staff^†^	3 (33.3)	6 (46.2)	5 (33.3)	2 (20.0)	14 (37.8)
Post-graduate students	0	0	3 (20.0)	2 (13.3)	3 (8.1)
Program managers and coordinators	6 (66.7)	5 (38.5)	3 (20.0)	8 (53.3)	14 (37.8)
Research staff	0	2 (15.4)	2 (13.3)	3 (20.0)	4 (10.8)
University lecturers/tutorial assistants	0	0	2 (13.3)	3 (20.0)	2 (5.4)
**Employer of trainees**					
Partners In Health/Rwanda	5 (55.6)	7 (53.8)	5 (33.3)	6 (40.0)	23 (44.2)
Rwanda Ministry of Health	4 (44.4)	6 (46.2)	5 (33.3)	3 (20.0)	18 (34.6)
University of Rwanda	0	0	5 (33.3)	3 (20.0)	8 (15.4)
Rwanda Biomedical Centre	0	0	0	3 (20.0)	3 (5.8)
**Research topic selection**					
First choice topic/trainee proposed topic	9 (100.0)	9 (69.2)	13 (86.7)	14 (93.3)	45 (86.5)
Second choice topic	n/a	2 (15.4)	1 (6.7)	1 (6.7)	4 (7.7)
Topic assigned	n/a	2 (15.4)	1 (6.7)	0	3 (5.8)
**Research proposals**	**5**	**7**	**8**	**8**	**28**
Manuscripts published	5 (100.0)	7 (100.0)	8 (100.0)	7 (87.5)	27 (96.4)
Manuscripts under-review	0	0	0	0	0
Manuscripts under-development	0	0	0	1 (12.5)	1 (3.6)
**Conference/meeting presentations^††^**	**3**	**3**	**18**	**6**	**30**
Study facility	0	0	13 (72.2)	0	13 (43.3)
Rwanda conference	3 (100.0)	2 (66.7)	3 (16.7)	4 (66.7)	12 (40.0)
International conference	0	1 (33.3)	2 (11.1)	2 (33.3)	5 (16.7)
**Research capacity outcomes**					
Trainee become junior or project mentor	0	1	1	1	3
Junior mentor become facilitator/mentor	1	1	0	0	2
Trainee enroll in graduate research training	2	3	2	1	8

^†^ Included general practitioners, nurses, physiotherapists, pharmacists; This is captured in the text part of results. ^††^ Includes oral and poster presentations. Some projects were presented to >1 conference; In 2016, all projects were presented to the relevant facilities of study and we counted individuals project presentation; IORT: Intermediate Operational Research Course.

**Table 4:** Research projects completed in the four offerings of the intermediate operational research course in Rwanda.

Theme	Title of a research project	Year of training	Journal, year of publication

Infectious disease	A novel combined mother-infant clinic to optimize post-partum maternal retention, service utilization, and linkage to services in HIV care in rural Rwanda	2015	International Journal of Maternal Child Health and AIDS, 2017
	Adherence to renal function monitoring guidelines for HIV-infected patients on tenofovir-based antiretroviral therapy in rural Rwanda	2013	Africa Journal of AIDS and HIV Research, 2016
	Dental caries management at a rural district hospital in northern Rwanda: a neglected disease	2013	Public Health Action, 2015
Maternal child health	A retrospective review of the Pediatric Development Clinic implementation: a model to improve medical, nutritional and developmental outcomes of at-risk under-five children in rural Rwanda	2016	BMC Maternal Health, Neonatology and Perinatology, 2017
	Assessing retention in care after 12 months of the Pediatric Development Clinic implementation in rural Rwanda: a retrospective cohort study	2016	BMC Pediatrics, 2018
	Bubble CPAP to support preterm infants in rural Rwanda: a retrospective cohort study	2013	BMC Pediatrics, 2015
	Care seeking patterns of families that experienced under-five deaths in rural Rwanda	2015	PLoS One, 2018
	A retrospective study of neonatal case management and outcomes in rural Rwanda post implementation of a national neonatal care package for sick and small infants	2015	BMC Pediatrics, 2018
	Developmental outcomes of preterm/low birth weight toddlers and term peers in Rwanda	2017	Annals of Global Health, 2020
	High burden of undernutrition among at-risk children in neonatal follow-up clinic in Rwanda	2017	Annals of Global Health, 2020
Non-communicable disease	Pregnancy-associated breast cancer in rural Rwanda: The experience of Butaro Cancer Center of Excellence	2016	BMC Cancer, 2018
	Integration of Chronic Oncology Services in Noncommunicable Disease Clinic in Rural Rwanda	2016	Annals of Global Health, 2020
	Treating persistent asthma in rural Rwanda: Characteristics, management, and 24-month outcomes	2015	International Journal of Tuberculosis and Lung Disease, 2017
	A clinical mentorship and quality improvement program to support health center nurses manage type 2 diabetes in rural Rwandan settings	2016	Journal of Diabetes Research, 2017
	Factors associated with loss to follow-up among cervical cancer patients at Butaro Cancer Center of Excellence, 2012–2017	2017	Annals of Global Health, 2020
	Treatment details and outcomes of Kaposi Sarcoma patients treated with paclitaxel at Butaro Cancer Center of Excellence, Rwanda, 2012–2017	2017	Under-development
Surgery	Assessing the cost of laparotomy at a rural district hospital in Rwanda using time-driven activity-based costing	2016	BJS Open, 2018
	Longer travel time to district hospital worsens neonatal outcomes: a retrospective cross-sectional study of the effect of delays in receiving emergency cesarean section in Rwanda	2016	BMC Pregnancy and Childbirth, 2017
	Maternal predictors of neonatal outcomes after emergency cesarean section: A retrospective study in three rural district hospitals in Rwanda	2016	BMC Maternal Health, Neonatology and Perinatology, 2017
	Non-obstetric surgical care at three rural district hospitals in Rwanda: more human capacity and surgical equipment may increase operative care	2015	World Journal of Surgery, 2016
	Emergency general surgery in Rwandan district hospitals: a cross-sectional study of spectrum, management, and patient outcomes	2016	BMC Surgery, 2017
	Referral patterns and predictors of referral delays for patients with traumatic injuries in rural Rwanda	2015	Surgery, 2016
	Presentation of Paediatric unintentional injuries at rural hospitals in Rwanda: A retrospective study	2017	Annals of Global Health, 2020
	Perioperative management and outcomes following cesarean section – a cross-sectional study from rural Rwanda, 2017	2017	Journals of Surgical Research, 2019
	Postoperative rheumatic heart disease follow-up: creating a national registry and first results from Rwanda	2017	Annals of Global Health, 2020
Supply chain	Assessment of essential medicines stock-outs at health centers in Burera District in Northern Rwanda	2013	Rwanda Journal of Medicine and Health Sciences, 2015
	Assessing prescribing patterns of essential medicines in three rural district hospitals in Rwanda	2013	International Journal of Pharmacy, 2015
	Caring for NCD patients in rural Africa: A descriptive study of the availability, costs and stock-outs for essential drugs in three rural districts in Rwanda	2017	Annals of Global Health, 2020

HIV: Human immunodeficiency virus; CPAP: Continuous positive airway pressure; NCD: Non communicable diseases.

Over the four courses, IORT trainees developed 28 research proposals and manuscripts (Table 3). As of July 2020, 96.4% (n = 27) of these projects were published in peer-reviewed journals, and one (3.6%) was under-development. 12 of the 53 trainees (22.6%) disseminated their research findings in regional or international conferences in Rwanda, and five trainees (9.4%) disseminated their findings at international conferences in Canada and the United States. Three former trainees have since become junior or project mentors in the course, and two junior mentors have become either a project mentor or a senior course mentor. Post-course, eight trainees (15.1%) enrolled in masters-level research training programs.

### Course evaluation

The majority of 2016 applicants (N = 36) (69.4%, n = 25) had previously participated in a research project, often as an academic thesis (Table 5). Nearly half (47.2%, n = 17) had completed short research-related courses in epidemiology or biostatistics, and 13.9% (n = 5) had completed PIH/Rwanda’s Introduction to Research Course [16]. By their reports, course applicants’ goals varied from improving their research skills (83.3%, n = 30) to enhancing their ability to identify and close practice gaps (75.0%, n = 27) and improving their ability to critically consume scientific literature (38.9%, n = 14).

**Table 5:** Training evaluation completed by the third cohort of IORT trainees.

	n	%	Median LC^†^

**Pre-training evaluation completed by 2016 applicants N = 36**			
**Trainee background during course application**			
Participation in a research project	25	69.4	
Completion of research related short course	17	47.2	
Presentation at local meetings	11	30.6	
Data collection and management	8	22.2	
Participation in a research writing workshop	7	19.4	
Completion of Introduction to Research course by PIH/Rwanda	5	13.9	
Member of a Research committee or research journal	4	11.1	
Research ethics training	3	8.3	
Data analysis	2	5.6	
Mentoring students on research skills	2	5.6	
**Trainee motivations for course application**			
Improve research skills	30	83.3	
Enhance the ability to identify and close practice gaps	27	75.0	
Critical consumption of scientific literature	14	38.9	
Become a research leader	13	36.1	
For professional development	7	19.4	
Improve ability to mentor or teach research skills	7	19.4	
Effective participation in research projects	6	16.7	
Improve the use of existing data	5	13.9	
**Mid-course trainee reaction completed 2016 trainees N = 15**			
Training content was interesting	15	100.0	4
Training content was relevant	13	86.7	4
Training did not cover all concepts	6	40.0	2
Training improved my research skills	14	93.3	4
Training improved my analytical skills	13	86.7	4
The facilitators were engaging	14	93.3	4
The facilitators were knowledgeable	15	100.0	4
The training team was knowledgeable	13	86.7	4
The training team was not available to help	10	66.7	1
Training pace was just right	13	86.7	2
**I would recommend this training to my colleagues because it^††^**	13	86.7	
Builds research skills‡	14	93.3	
Provides Stata to facilitate future research projects	9	60.0	
Improves critical research consumption	7	46.7	
Uses learner-centered learning by doing methodology	4	26.7	
**Training should improve on**			
In-person and practicum time for data analysis	8	53.3	
More training time for data management	5	33.3	
Increasing in-person days per session to 7–8 days	3	20.0	

IORT: Intermediate Operational Research Training; ^††^Two participants did not respond to this question. ‡ Skills included critical thinking, writing, analytical skills, research production, mentorship, and leadership. ^†^ Likert scores ranged from strongly agree (score = 4), agree (score = 3), disagree (score = 2) and strongly disagree (score = 1) or too fast (score = 3), just right (score = 2) and too slow (score = 1).

In the mid-course evaluation, trainees reported improvement in their research (93.3%, n = 14) and analytical (86.7%, n = 13) skills (Table 5). Most trainees strongly agreed that facilitators were engaging (93.3%, n = 14), and most agreed that the training team was available to support them (66.7%, n = 10). Many trainees (86.7%, n = 13) would recommend the course because IORT builds research skills (93.3, n = 14) covering skills such as critical thinking, writing, research production, mentorship, and leadership. The trainees recommended an increase in in-person and practicum time for data analysis (53.3%, n = 8) to ensure the finalization of results before the manuscript development training.

### Course costs (US$)

For individual trainees to complete the course, we spent a median cost of $908 (Range $739–$1,253) per trainee, excluding the cost of laptops which the trainees either owned or borrowed from PIH/Rwanda (Table 6). The median unit cost per research project was $6,653 ($4,023–$12,631) with data collection ($892, $643–$4,528), ethical review ($1,158, $500–$1,450), publication fees ($1,300, $755–$2,100), and international conference travels ($2,645, $1,500–$3,790) as the main cost drivers.

**Table 6:** Cost of designing and delivering an operational research course with research project implementation from research idea to publication in Rwanda.

	Median and (Range) for unit costs (US$)	Median and (Range) for total costs (US$)^†^

**Trainee costs**		
Local travel per participant*	57 (25–100)	1257 (550–2200)
Training meals per participant	261 (193–358)	5736 (4242–7865)
Room and board per participant	236 (212–382)	5188 (4656–8404)
Printing training materials per participant	23 (21–40)	505 (455–880)
Communication (airtime and data) per trainee	103 (83–133)	1648 (1321–2124)
Supplies per trainee		
Data analysis software (Stata)	200 (200–213)	3200 (3200–3408)
Flash drives	28 (6–28)	448 (96–449)
Laptops^ǂ^	500 (500–500)	8000 (8000–8000)
**Sub-total**	**1408 (1239–1753)**	**25982 (22520–33330)**
**Sub-total (excluding laptops)^ǂ^**	**908 (739–1253)**	17982 **(14520–25330)**
**Research project costs**		
Data collection per project	892 (643–4528)	7139 (5141–36224)
Training data collectors per course	234 (194–274)	234 (194–274)
Ethical review per protocol	1158 (500–1450)	9267 (4000–11600)
Local/regional conference travel per project	300 (300–300)	2400 (2400–2400)
Research meetings per project	123 (111–189)	986 (891–1515)
Publication fee per project^ǂ^	1300 (775–2100)	10400 (6200–16800)
International conference travel per project^ǂ^	2645 (1500–3790)	21160 (12000–30320)
**Sub-total**	**6653 (4023–12631)**	**51585 (30826–99133)**
**Sub-total (excluding publication fee and international conference travel)^ǂ^**	**2708 (1748–6741)**	**20025 (12626–52013)**
**Training delivery and mentorship costs**		
Training venue and equipment per course	1035 (1032–1111)	1035 (1032–1111)
Research administrative support per course	4438 (4438–4438)	4438 (4438–4438)
Senior course mentor (20% time)	21770 (17847–25693)	43540 (35694–51386)
Training coordinator (50% time)^§^	14234 (5176–23291)	14234 (5176–23291)
Junior mentor (20% time)^ǂ^	5693 (2070–9316)	17079 (6210–27948)
International travel per US-based mentor^ǂ^	**6970 (6541–7273)**	**13940 (13082–14546)**
**Sub-total**	**54140 (37104–71122)**	**94265 (65633–122720)**
**Sub-total (excluding international travels for mentors, and paid junior mentors)^ǂ^**	**47170 (30563–63849)**	**63247 (46340–80226)**
**Total costs**		**171833 (118978–255183)**
**Total cost excluding laptops, publication fees, international conference travel, international travel for mentors and paid junior mentors^ǂ^**		**101254 (73486–157569)**

^†^ This includes a total of 22 participants – 16 trainees paired to implement 8 research projects, with 2 senior course mentors and 4 junior mentors, one of whom also functions as the training coordinator. * The cost of travel per participant is sensitive to the design of the course – a seven 2-day sessions course has high travel cost compared to a course of three 1-week sessions; ^ǂ^ Laptops, publication fees, international conference travels, international travels for US-based mentors, and junior mentors were considered avoidable direct costs. Trainees used their own laptops or borrowed from PIH/Rwanda, we sought publication fee waivers and scholarships for international conference travels, we prioritized Rwanda-based mentors where possible and junior mentors time, except for training coordinator, was in-kind support provided through collaborating research teams; ^§^ Training coordinator also functioned as a junior mentor.

The bulk of total training costs fell under training delivery and mentorship ($63,247, $46,340–$80,226) (Table 6). This was at least three times the trainee costs ($17,982, $14,520–$25,330) and the research project costs ($20,025, $12,626–$52,013). These estimates excluded the costs of international conference travels, publication fees, international travels for mentors, as well as junior mentors’ time which was provided in kind. The total cost for a three one-week IORT courses in Rwanda was $101,254 ($73,486–$157,569) with the above-mentioned exclusions. However, the overall cost without exclusions increased to $171,833 ($118,978–$255,183).

## Lessons and experiences from the four IORT courses

### Course completion rates

The high course completion rates in the IORT is comparable to the course completion rates for the SORT IT courses (~89%) [30,31]. We attribute this success to both the training approach and the intensive, ongoing hands-on mentorship. The learning-by-doing approach and the deliverable-driven model reinforced learning and task orientation for both trainees and mentors [17,24,32]. The practical nature of the learning-by-doing approach demonstrated the value of the course to both trainees and their institutions through publications and the development of work-related skills in critical thinking, writing, and analysis. The intensive, ongoing hands-on mentorship strengthened learning outcomes and availed the necessary support for the completion of training milestones, similar to what has been observed in other research capacity building programs [20,21,22,23,24,33]. It also reduced the chances of trainees abandoning research projects due to work pressures, especially during the practicum period. Three trainees who changed employers within Rwanda during the course received support from their new employer to complete the course, underscoring trainee commitment and the value of research skills in Rwanda [13].

### Gender distribution

The progressively more gender equitable trend in the course reflects programmatic decisions to intentionally select more qualified women in the course. In addition, the proportion of female applicants increased from 4.2% (n = 1) in 2013 to 36.4% (n = 16) in 2017. However, we still had more male than female trainees. This gender imbalance, also observed by other research capacity-building programs, may be attributed partly to time-demanding family obligations that typically fall on women in this context [16,33,34,35]. Of note, nevertheless, the course completion rates were the same for female and male trainees, as has been noted in other training models [16,36]. To promote gender equity in similar courses, we recommend allocating training spaces for female trainees. Furthermore, implementers should cultivate a family-friendly training environment where trainees can bring their children, as was made available for IORT trainees [33,36]. Committing to long-term research engagement through sustaining mentorship relationships may increase the number of female trainees in research [33,36].

### Partnership development

Over time, our course increased both institutional and inter-professional partnerships, which may promote future research and build research capacity in Rwanda. In our experience, the diversity in trainees’ professional and institutional backgrounds enhanced knowledge and skills sharing among trainees. It also advanced the formation of a national network of Rwandan researchers. Trainees such as university lecturers have a greater potential for mentoring new students in research skills through their academic positions. We postulate that an annual meeting of a national network of IORT alumni and trainees may sustain these collaborations. Such a meeting should target the growth of additional research leadership skills such as grant writing. We plan to explore this in the future.

### Dissemination

There is a growing call for systematic evaluation of the impact of courses like IORT [15,37]. Individual research capacity building courses often measure research outputs such as publications and short- and long-term assessments of the course’s impact on trainees’ ability to use and transfer research skills [37]. We measured peer-reviewed publications and conference attendance, both of which demonstrate the feasibility of research leadership by first-time researchers in rural and low-income settings. Trainees disseminated their research findings at the three district health facilities where their studies were conducted. This encouraged the uptake of operational research findings by health providers. Strategies to enhance the impact of local dissemination of results such as outlining practice briefs and monitoring the uptake of research results are needed [1]. While we did not measure the link between operational research and policy or practice, similar courses such as SORT IT have demonstrated this relationship [38].

### Trainee experiences

Through the surveys, trainees critically reflected on the course, highlighting the short-term impact of the IORT course in improving their critical thinking, writing, and data analysis skills [39]. The availability of the mentorship team with a commitment to providing hands-on intensive mentorship improved learning outcomes. Combined with the first author publications, these are early indications of the impact of the course on trainees’ professional development. Future evaluations need to be more systematic and could assess learning acquisition and behavior change as has been shown in the SORT IT course [28,30,36].

### Training costs and funding

Few research training programs report their costs. For those that do, the costs vary from US$500 to US$20,000 per research project depending on the competencies covered, length of training, and location of training participants [3,26]. These costs focus on research project costs, excluding training and mentorship costs. In contrast, our paper describes the cost of delivering an operational research course with details on training, mentorship, and research project costs. These cost estimates show that while intensive hands-on mentorship is crucial for the success of IORT, it is costly. Costs for similar training programs will be program and country-specific. Our estimates provide a rational basis for budgeting and fundraising for other implementers and funders planning for similar trainings.

To fund this course, we leveraged institutional resources and external partnerships. Institutionally, we relied on existing resources of PIH/Rwanda, including training facilities and vehicles for field travels. PIH/Rwanda also funded three of the four courses through operational funds and innovation grants. PIH/Rwanda’s partnership with Harvard Medical School’s (HMS) Global Health Research Core provided in-kind course delivery and mentorship support. HMS funded the time for one senior course mentor for all four courses, another senior course mentor for two courses, and one training coordinator/junior mentor for three courses. We also received a grant through Harvard Global Health Initiative Burke Global Health Fellowship Grant for the 2016 course. Project mentors with their project-affiliated junior mentors also provided in-kind mentorship, estimated at 5% and 10% of their time, respectively. We did not estimate the cost for project mentors given the diversity of these mentors.

### Other challenges and limitations

Our reliance on in-kind support for course delivery and mentorship, a key element of the course, affects the sustainability of IORT, especially in a context of declining grant support for international training. This is critical for IORT approaches such as learning-by-doing and intensive hands-on mentorship that are both resource and labor intensive. For example, we fully sponsored trainees, including providing research tools such as analytical software to make the learning-by-doing approach feasible. Our senior mentors experienced a high mentorship workload, which makes senior mentors’ role unsustainable without dedicated funding. We hope that the outcomes of this course encourage funders to invest more in mentorship-oriented capacity-building approaches. We also encourage the government of Rwanda to adopt IORT and dedicate funding for sustaining and scaling the program in Rwanda.

Although a key output for the training is a peer-reviewed publication, publication fees were often prohibitive for the course, as has been observed by others [40]. We prioritized publishing in open access journals that provide publication fee waivers to ensure our training papers were publicly available for all Rwandans and other LMIC users. We also recommend the WHO’s HINARI program, which helped our trainees access articles published in closed-access journals. Similarly, international conference travels were cost-prohibitive. As such, we encouraged trainees to attend local and regional conferences in Africa and to apply for scholarships to attend international conferences.

We only evaluated our course with the 2016 cohort due to challenges with prior planning and recognizing the need for a system of evaluation on a course-by-course basis. Equally, a system for follow-up with the trainees on research engagement post-course, as well as tracking the uptake of research project results on practice and policy, would further demonstrate the value of IORT. This should also measure research capacity outcomes such as changes in publication output post IORT, access to research grants, and growth of trainees and junior mentors to research roles.

## Conclusion

IORT aims to develop a pool of Rwandan researchers who become critical and effective health research leaders and collaborators in Rwanda. Over the four years of this course, we have trained 53 health professionals who developed 28 research manuscripts, most of which have been published in peer-reviewed journals. We implemented the course iteratively, with annual reflections on what worked best and adaptations to address new and ongoing challenges. We believe there is ‘more than one way to slice the cake’ [32] when it comes to these courses, and every implementing organization should be sensitive to its context, open to learn, and flexible. In our case, the success of the course depended on the learning-by-doing approach and the intensive hands-on mentorship, albeit with high costs. Long-term partnership with HMS provided in-kind course delivery and mentorship support. Trainees found the course enriching in their professional development. We encourage funders to prioritize mentorship-oriented capacity-building initiatives, as they are a pathway to strengthening national health research systems in LMICs and reducing inequities in global health research leadership and partnerships.
